# Fine Analysis of Lymphocyte Subpopulations in SARS-CoV-2 Infected Patients: Differential Profiling of Patients With Severe Outcome

**DOI:** 10.3389/fimmu.2022.889813

**Published:** 2022-07-15

**Authors:** Giovanna Clavarino, Corentin Leroy, Olivier Epaulard, Tatiana Raskovalova, Antoine Vilotitch, Martine Pernollet, Chantal Dumestre-Pérard, Federica Defendi, Marion Le Maréchal, Audrey Le Gouellec, Pierre Audoin, Jean-Luc Bosson, Pascal Poignard, Matthieu Roustit, Marie-Christine Jacob, Jean-Yves Cesbron

**Affiliations:** ^1^ Laboratoire d’Immunologie, Pôle de Biologie, Centre Hospitalier Universitaire Grenoble Alpes, Grenoble, France; ^2^ Cellule d’Ingénierie des Données, Centre Hospitalier Universitaire Grenoble Alpes, Grenoble, France; ^3^ Centre d’Investigation Clinique de l’Innovation et de la Technologie (CIC-IT), Centre Hospitalier Universitaire Grenoble Alpes, Grenoble, France; ^4^ Service de Maladies Infectieuses, Centre Hospitalier Universitaire Grenoble Alpes, Grenoble, France; ^5^ Univ. Grenoble Alpes, CNRS, Grenoble INP, TIMC, Grenoble, France; ^6^ Univ. Grenoble Alpes, CNRS, CEA, Institut de Biologie Structurale, Grenoble, France; ^7^ Service de Biochimie Biologie Moléculaire et Toxicologie Environnementale, Pôle de Biologie, Centre Hospitalier Universitaire Grenoble Alpes, Grenoble, France; ^8^ Unité recherche, Pôle de Biologie, Centre Hospitalier Universitaire Grenoble Alpes, Grenoble, France; ^9^ Laboratoire de Virologie, Pôle de Biologie, Centre Hospitalier Universitaire Grenoble Alpes, Grenoble, France; ^10^ Centre d’Investigation Clinique INSERM CIC1406, Centre Hospitalier Universitaire Grenoble Alpes, Grenoble, France; ^11^ Univ. Grenoble Alpes, INSERM, UMR 1300, HP2, Grenoble, France

**Keywords:** SARS-CoV-2, COVID-19, lymphocytes, flow cytometry, disease severity

## Abstract

COVID-19 is caused by the human pathogen severe acute respiratory syndrome coronavirus 2 (SARS-CoV-2) and has resulted in widespread morbidity and mortality. CD4^+^ T cells, CD8^+^ T cells and neutralizing antibodies all contribute to control SARS-CoV-2 infection. However, heterogeneity is a major factor in disease severity and in immune innate and adaptive responses to SARS-CoV-2. We performed a deep analysis by flow cytometry of lymphocyte populations of 125 hospitalized SARS-CoV-2 infected patients on the day of hospital admission. Five clusters of patients were identified using hierarchical classification on the basis of their immunophenotypic profile, with different mortality outcomes. Some characteristics were observed in all the clusters of patients, such as lymphopenia and an elevated level of effector CD8^+^CCR7^-^ T cells. However, low levels of T cell activation are associated to a better disease outcome; on the other hand, profound CD8^+^ T-cell lymphopenia, a high level of CD4^+^ and CD8^+^ T-cell activation and a high level of CD8^+^ T-cell senescence are associated with a higher mortality outcome. Furthermore, a cluster of patient was characterized by high B-cell responses with an extremely high level of plasmablasts. Our study points out the prognostic value of lymphocyte parameters such as T-cell activation and senescence and strengthen the interest in treating the patients early in course of the disease with targeted immunomodulatory therapies based on the type of adaptive response of each patient.

## Introduction

Coronavirus disease 2019 (COVID-19) is caused by the human pathogen severe acute respiratory syndrome coronavirus 2 (SARS-CoV-2) and has resulted in widespread morbidity and mortality. The total number of lymphocytes, CD4^+^ T cells, CD8^+^ T cells and natural killer cells significantly decreases in COVID-19 patients, with the lowest levels in severe cases (1); in particular, a decrease in CD8^+^ T cells and an increase in plasmablasts in infected patients have been described (2, 3). COVID-19 severity and duration seem to be dependent on the early evasion of innate immune recognition, especially with defects in type 1 interferon pathways (2, 4, 5), and the subsequent kinetics of the adaptive immune response (6). Since severe COVID-19 is associated with high levels of IL-6, sepsis has been used as a prototype of critical illness for the understanding of severe COVID-19 pathogenesis. However, even if a low expression of human leucocyte antigen D related (HLA-DR) on CD14^+^ monocytes has been described in some patients, this pattern is distinct from the immunoparalysis state reported in bacterial sepsis or severe respiratory failure caused by influenza (7, 8).

CD4^+^ T cells, CD8^+^ T cells and neutralizing antibodies all contribute to control SARS-CoV-2 infection. However, heterogeneity is a major factor in disease severity and in immune innate and adaptive responses to SARS-CoV-2. Deep immune profiling of lymphocyte populations has been performed by using high dimensional flow cytometry, leading to the definition of different immunotypes: immunotype 1 characterized by CD4^+^ T cell activation, exhausted CD8^+^ T cells, presence of plasmablasts and associated with more severe disease, immunotype 2 characterized by less CD4^+^ T cell activation, the presence of effector CD8^+^ subsets and proliferating memory B cells, and immunotype 3 with minimal lymphocyte activation response and negatively associated with disease severity (9, 10). In another study conducted with principal components analysis and hierarchical clustering, a vast array of immunological parameters has been measured, with the description of three distinct phenotypes: a humoral response deficiency phenotype, a hyper-inflammatory phenotype and a complement-dependent phenotype (11). In the present study, we performed a fine analysis of lymphocyte subsets of SARS-CoV-2 infected hospitalized patients on the day of admission in order to better characterize the adaptive immune response and possibly define patient trajectories with different disease progression courses.

## Materials and Methods

### Ethics Statement

Overall 146 patients infected with SARS-CoV-2 were recruited in Grenoble Alpes University Hospital between March and September 2020, either in a retrospective (n=127) or in a prospective (n=19) study. Clinical and biological data were fully available for 125 patients (**Supplementary Figure 1**). The study was performed in accordance with the Declaration of Helsinki, Good Clinical Practice guidelines and CNIL (Commission Nationale de l’Informatique et des Libertés) methodology reference. Patients were informed and non-opposition (BioMarCoViD retrospective study) or written consent (AcNT-COVID-19 prospective study) was obtained, according to French law. Prospective study was approved by the relevant local ethics committee (N°IDRCB: 2020-A00904-35) and registered in clinicaltrial.gov (NCT04596098).

Laboratory confirmation for SARS-CoV-2 was defined as a positive result of real-time reverse transcriptase-polymerase chain reaction assay of nasopharyngeal swabs. Follow-up of the patients at days 3, 7 and 13 was carried out, and clinical data, oxygen requirements, intensive care unit (ICU) admission, steroid treatment, and laboratory data were collected for each time point; patients were classified in severity classes on the basis of oxygen requirement, ICU admission, limitation of therapeutic effort and mortality (**Supplementary Table 1**), as in (12).

### Flow Cytometric Peripheral Blood Lymphocyte Analysis

Peripheral blood samples were collected in EDTA-containing tubes (Becton Dickinson). Cell staining was performed on whole blood samples using a direct immunofluorescence method with erythrocytes lysis and washing. Cells were stained with a panel of four 8-colour antibody combinations (**Table 1**). Clone and isotypes are detailed in **Supplementary Table 2**. The antibodies were used at the dilution recommended by the manufacturers. Acquisition was performed using BD FACSCanto-II flow cytometer (BD Biosciences, San José, CA) and analysis done with BD FACSDiva 8 software (BD Biosciences, San José, CA). The absolute numbers of subsets were calculated by multiplying their percentage by the total lymphocyte number obtained from an ABX MICROS 60 device (HORIBA ABX SAS, Montpellier, France). BD CompBeads (BD Biosciences) were used for compensation settings. Cytometer performances were checked daily using CS&T IVD beads (BD Biosciences). Gating strategy for lymphocyte subsets analysis is shown in **Supplementary Figure 2**. Immunophenotype of cell subsets is detailed in **Table 2**.

**Table 1:** Panel of four antibody combinations used in the study.

Fluoro-chrome	FITC	PE	PerCP-Cy5.5	PE-Cy7	APC	APC-Cy7 or APC-H7	V450/BV421	V500
Tube								
1	CD3	CD56 and CD16	CD45	CD4	CD19	CD8	HLA-DR	x
2	CD57	CD8	CD4	CD3	CD45RA	x	CCR7	x
3	CD3	CD127	CD4	CD56	CD25	CD16	CD7	CD45
4	IgD	CD10	CD38	CD27	IgM	CD19	x	x

**Table 2:** Cell subsets and corresponding immunophenotypes.

Cell subset	Immunophenotype
**T-cell subsets**	
Total CD4^+^ T cells	CD3^+^ CD4^+^
Naive CD4^+^ T cells	CD45RA^+^ CCR7^+^
Central memory CD4^+^ T cells	CD45RA^-^ CCR7^+^
Effector CD4^+^ T cells	CD45RA^+/-^ CCR7^-^
Regulatory T cells	CD4^+^ CD127^low^ CD25^high^
Total CD8^+^ T cells	CD3^+^ CD8^+^
Naive CD8^+^ T cells	CD45RA^+^ CCR7^+^
Central memory CD8^+^ T cells	CD45RA^-^ CCR7^+^
Effector CD8^+^ T cells	CD45RA^+/-^ CCR7^-^
**B-cell subsets**	
Total B cells	CD19^+^
Transitional B cells	IgD^+^ CD27^-^ CD10^+^ CD38^high^
Naive B cells	IgD^+^ CD27^-^ CD10^-^ CD38^low^
Natural memory B cells	IgD^+^ CD27^+^
Post germinal memory B cells	IgD^-^ CD27^+^ CD38^low^
Plasmablasts	IgD^-^ CD27^high^ CD38^high^
**NK cells**	
Total NK cells	CD56^+^ or CD16^+^ and CD3^-^
Cytotoxic NK cells	CD56^+^ CD16^+^ CD3^-^
Immunomodulatory NK cells	CD56^-^ CD16^+^ CD3^-^
Inflammatory NK cells	CD56^+^ CD16^-^ CD3^-^
**Monocytes**	
Total monocytes	CD45^high^ SSC^intermediate^
Non-conventional monocytes	CD16^+^

### Flow Cytometric Monocyte HLA-DR Expression Analysis

Peripheral blood samples were collected in EDTA-containing tubes which were kept on ice and rapidly routed to the laboratory. Whole blood (50 µl) was stained with 20 µl of QuantiBrite anti-HLA-DR/Monocyte mixture (QuantiBrite anti-HLA-DR PE (clone L243)/Anti-monocytes (CD14) PerCP-Cy5.5 (clone MΦP9), Becton Dickinson, San José, CA) at room temperature for 30 min in the dark. Samples were the lysed using the FACS Lysing solution (Becton Dickinson) for 15 min. After a washing step, cells were analyzed with BD FACSCanto-II flow cytometer and FACSDiva software version 8 (BD Biosciences, San José, CA). Monocytes were first gated out from other cells on the basis of CD14 expression and mHLA-DR expression was then measured on their surface (mono-parametric histogram) as median of fluorescence intensity related to the entire monocyte population (as recommended by manufacturer). These results were then transformed in AB/C (number of antibodies fixed per cell) thanks to calibrated PE-beads (BD QuantiBrite-PE Beads, Becton Dickinson).

### Statistical Analysis

Hierarchical ascendant cluster analysis with the Ward method (13, 14) was used to identify groups of SARS-CoV-2 infected patients on the basis of immunophenotypic profiling. Immunophenotypic parameters were the following: leucocytes G/L, lymphocytes G/L, total CD3^+^ T cells G/L, total CD4^+^ T cells G/L, total CD8^+^ T cells G/L, total CD3^+^ T cells %, total CD4^+^ T cells %, total CD8^+^ T cells %, CD4^+^/CD8^+^, CD3^-^CD56^+^ %, naive CD4^+^ T cells %, central memory CD4^+^ T cells %, effector CD4^+^ T cells %, naive CD8^+^ T cells %, central memory CD8^+^ T cells %, effector CD8^+^ T cells %, CD8 CDRA^+^ CCR7%, regulatory T cells %, CD4^-^CD8^-^/CD3^+^ %, CD57^+^/CD4^+^ %, CD57^+^/CD8^+^ %, CD57%, CD56^+^/CD4^+^ %, CD56^+^/CD8^+^ %, HLA-DR^+^/CD4^+^ %, HLA-DR^+^/CD8^+^ %, CD25^+^/CD4^+^ %, CD25^+^/CD8^+^ %, total B cells G/L total B cells %, transitional B cells %, naive B cells %, natural memory B cells %, post germinal memory B cells %, plasmablasts %, post germinal switched memory B cells %, total NK cells G/L, total NK cells %, cytotoxic NK cells %, inflammatory NK cells %, immunomodulatory NK cells %, CD16^-^ CD56^-^ %, total monocytes %, non-conventional monocytes %. We added age, which might be a variation factor for some of the lymphocyte subpopulations (15). Analysis was performed using Stata 15 (StataCorp, College Station, TX, USA); the optimal number of clusters was chosen using selection criterions of Calinski-Harabasz and Duda-Hart. Biological meaning of the clusters was analyzed by screening the values of every parameter, between all clusters. ANOVA F-test was conducted for parameters with a Gaussian distribution, and a non-parametric Mann-Whitney test was run for other distributions. For significant results, a specific cluster by cluster tests were conducted to identify the significantly different cluster. This was conducted using Student tests for gaussian distributions.

## Results

### Study Population and Lymphocyte Subset Analysis

Median age of the patients was 70 years (IQR [56-78]), and most were male (n=74, 59%); median body mass unit (BMI) was 27 kg/m^2^ (IQR [23-32]. Mean time from symptom onset to hospital admission was 11 (sd 5.6) days. Overall, 68 (54.4%) patients were classified as severe, 41 (32.8%) have been admitted to ICU during their follow-up and 14 (11.2%) deceased; 51 (40.8%) patients were treated with corticosteroids during the follow-up (**Table 3**).

**Table 3:** Description of the population.

	Study population
n	125
Men, n (%)	74 (59%)
Women, n (%)	51 (41%)
Time from symptom onset to first biological sample: mean (sd)	11 (5.6)
Age: median [IQR]	70 [55.6; 78.5]
BMI: median [IQR]	27.1 [23.3; 31.9]
CRP: median [IQR]	61 [25; 133]
Mortality, n (%)	14 (11.2%)
Severe COVID19^1^, n (%)	68 (54.4%)
ICU admission, n (%)	41 (32.8%)
Oxygen requirement, n (%)	87 (69.6%)
Limitation of therapeutic effort (LTE), n (%)	3 (2.4%)
Treated with corticosteroids, n (%)	51 (40.8%)

IQR: interquartile range.

^1^Severe Covid19 defined as: 0_2_<2L/min, ICU admission, LTE, decease.

The lymphocyte subpopulation analysis performed on the day of hospital admission is summarized in **Supplementary Table 3**. Lymphopenia was observed, with a median value of total lymphocyte count of 0.9 G/L (IQR [0.6-1.3]), affecting CD3^+^ cells (median 0.6 G/L [0.4-0.9]), CD4^+^ cells (median 0.4 G/L [0.2-0.6]), CD8^+^ cells (median 0.2 G/L [0.1-0.3]), NK cells (median 0.1 G/L [0.1-0.2]), and CD19^+^ cells (median 0.1 G/L [0.1-0.2]). Other characteristics found in the study population were: effector CD8^+^CCR7^-^ T cells above normal value (median 64% [49-81]), elevated levels of CD19^+^CD38^high^ plasmablasts (median 7% [3-18]), HLA-DR molecules on CD14^+^ monocytes (mHLA-DR) in normal ranges (median 35952 AB/C [19850-50973]) (5, 12, 13).

### Clusters of SARS-CoV-2 Infected Patients According to Immunophenotypic Profiling

**Figure 1:**
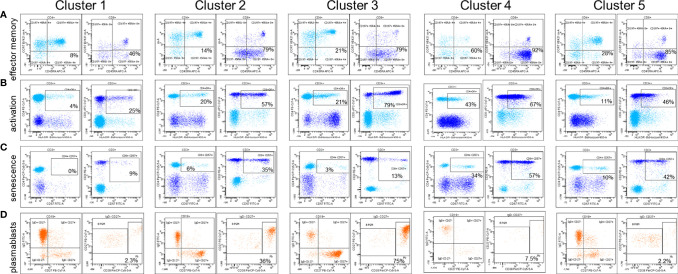
Examples of lymphocyte immunophenotypic characteristics for each cluster of patients. **(A)** Effector memory lymphocytes are defined as CD197- (CCR7-) CD45RA+/- within CD4+ and CD8+ T cells. **(B)** Activated lymphocytes within CD4+ and CD8+ T cells are defined by HLA-DR expression **(C)** Senescent lymphocytes within CD4+ and CD8+ T cells are defined by CD57 expression. **(D)** Plasmablasts are defined within CD19+ IgD- CD27+ cells as CD38high CD27high cells. B PGM, post germinal memory B cells; Pb, plasmablasts.

Five clusters of patients were identified regarding immunophenotypic profile (**Supplementary Figure 3**). Statistical analysis of each cellular subpopulation is reported in **Supplementary Table 4**; the main characteristics of patients in the different clusters are described in **Table 4** and **Figure 1**.

**Table 4:** Clusters of SARS-CoV-2 infected patients based on immunophenotypic profiling.

	Cluster 1	Cluster 2	Cluster 3	Cluster 4	Cluster 5	p-valueglobal test	p-value comparaison 2 by 2
**Population size**, n (%)	41 (33)	23 (18)	21 (17)	17 (14)	23 (18)		
**Age**, mean (sd)	56. (18.7)	67 (11.6)	70 (12.8)	72 (14.0)	79 (10.8)	p=0.0001^1^	p<0.0001^2^ cluster 1 against others; cluster 5 against others
**Lymphocytes G/L,** mean (sd)	1.1 (0.62)	1.0 (0.51)	0.9 (0.6)	1.1 (0.52)	0.9 (0.43)	0.27^1^	
**CD8^+^ G/L**, mean (sd)	0.2 (0.17)	0.2 (0.16)	0.2 (0.14)	0.4 (0.25)	0.2 (0.13)	p=0.0014^1^	p<0.0001^2^ cluster 4 against others
**Effector CCR7^-/^CD8^+^ %**, mean (sd)	43 (12.1)	64 (7.3)	67 (12.2)	85 (9.5)	84 (7.0)	p=0.0001^1^	p<0.0001^3^ cluster 1 against others; clusters 4 and 5 against others
**Effector CCR7^-/^CD4^+^ %,** mean (sd)	13 (6.5)	14 (5.1)	21 (16.2)	39 (13.8)	21 (10.6)	p=0.0001^1^	p<0.0001^2^ cluster 4 against others
**HLA-DR^+^/CD4^+^ %**, mean (sd)	7 (3.2)	11 (5.8)	17 (11.6)	16 (7.3)	14 (7.2)	p=0.0001^1^	p<0.0001^2^ clusters 3, 4 and 5 against others
**HLA-DR^+^/CD8^+^ %**, mean (sd)	21 (9.2)	34 (11.9)	49 (18.1)	37 (16.9)	48 (17.6)	p=0.0001^1^	p<0.0001^2^ clusters 3, 4 and 5 against others
**CD57^+^/CD4^+^ %**, mean (sd)	1.7 (1.7)	1.7 (1.2)	4.9 (7.9)	19.3 (13.7)	5.7 (5.3)	p=0.0001^1^	p<0.0001^2^ cluster 4 against others
**CD57^+^/CD8^+^ %**, mean (sd)	14.2 (6.4)	33.1 (12.2)	19.5 (11.9)	51.7 (10.6)	43.5 (12.7)	p=0.0001^1^	p<0.0001^2^ clusters 1 and 3 against others
**CD38^high^/CD19^+^ %**, mean (sd)	7.8 (7.5)	8.3 (6.9)	33.2 (21.7)	8.6 (9.5)	9.5 (8.8)	p=0.0001^1^	p<0.0001^2^ cluster 3 against others
**mHLA-DR AB/C^5^**, mean (sd)	42043 (17037) (n=13)	25217 (17484) (n=10)	21009 (9785) (n=8)	44608 (20302) (n=8)	47150 (19101) (n=11)	p=0.004^4^	p<0.0001^3^ clusters 2 and 3 against others

^1^Kruskal-Wallis equality-of-populations rank test.

^2^Two-sample Wilcoxon rank-sum (Mann-Whitney) test.

^3^Two-sample t test with unequal variances.

^4^ANOVA F-test.

^5^ mHLA-DR AB/C : HLA-DR expression on CD14^+^ monocytes (number of antibodies fixed per cell).

Some characteristics were observed in all the clusters, such as lymphopenia, an elevated level of effector CD8^+^CCR7^-^ T cells (with extremely high levels in clusters 4 and 5) and an elevated level of plasmablasts (with extremely high levels in cluster 3).

**Figure 2:**
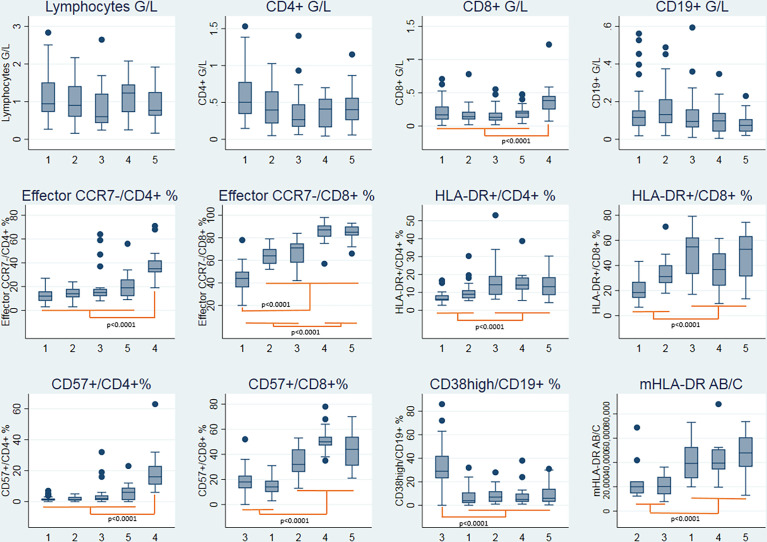
Boxplots representing the immunophenotypic characteristics of the five clusters of patients. Statistical analysis by hierarchical ascendant clustering discriminates the 125 patients with COVID-19 of the cohort in five distinct clusters according to the immunophenotypic variables (Supplementary Methods). Age was included in the model. Boxplots represent the median and the 25^th^ to 75^th^ percentiles, the whiskersrepresent the 10^th^ and the 90^th^ percentiles; outside values are represented by points.

Patients in cluster 1 were the youngest patients (mean age 56), with low T-cell activation (HLA-DR^+^/CD4^+^ mean 7% and HLA-DR^+^/CD8^+^ mean 21%) and very low T-cell senescence (CD57^+^/CD4^+^ mean 2% and CD57^+^/CD8^+^ mean 14%); no mortality was observed in this cluster. Patients of cluster 2 exhibited high T-cell activation (HLA-DR^+^/CD4^+^ mean 11% and HLA-DR^+^/CD8^+^ mean 34%) and high level of senescent T CD8^+^ cells (CD57^+^/CD8^+^ mean 33%). Cluster 3 was specifically characterized by an extremely elevated level of CD19^+^CD38^high^ plasmablasts (mean 33%); T-cell activation was very high (HLA-DR^+^/CD4^+^ mean 17%; HLA-DR^+^/CD8^+^ mean 49%), with very low level of senescent T cells (CD57^+^/CD4^+^ mean 2% and CD57^+^/CD8^+^ mean 18%). Cluster 4 was characterized by an extremely high level of effector CD8^+^CCR7^-^ T cells (mean 85%), a very high level of T-cell activation (HLA-DR^+^/CD4^+^ mean 16%; HLA-DR^+^/CD8^+^ mean 37%) and T-cell senescence (CD57^+^/CD4^+^ mean 19% and CD57^+^/CD8^+^ mean 52%). Similarly to cluster 4, cluster 5 was characterized by an extremely high level of effector CD8^+^CCR7^-^ T cells (mean 84%), a very high level of T-cell activation (HLA-DR^+^/CD4^+^ mean 14%; HLA-DR^+^/CD8^+^ mean 48%) and CD8^+^ T-cell senescence (CD57^+^/CD8^+^ mean 44%); patients in cluster 5 were the oldest (mean age 79). To note, cluster 4 was the only cluster characterized by normal levels of CD8^+^ cells (mean 0.4 G/L) and a high level of effector CD4^+^CCR7^-^ T cells (mean 39%) (**Table 4**; **Figures 1**, **2**).

**Figure 3:**
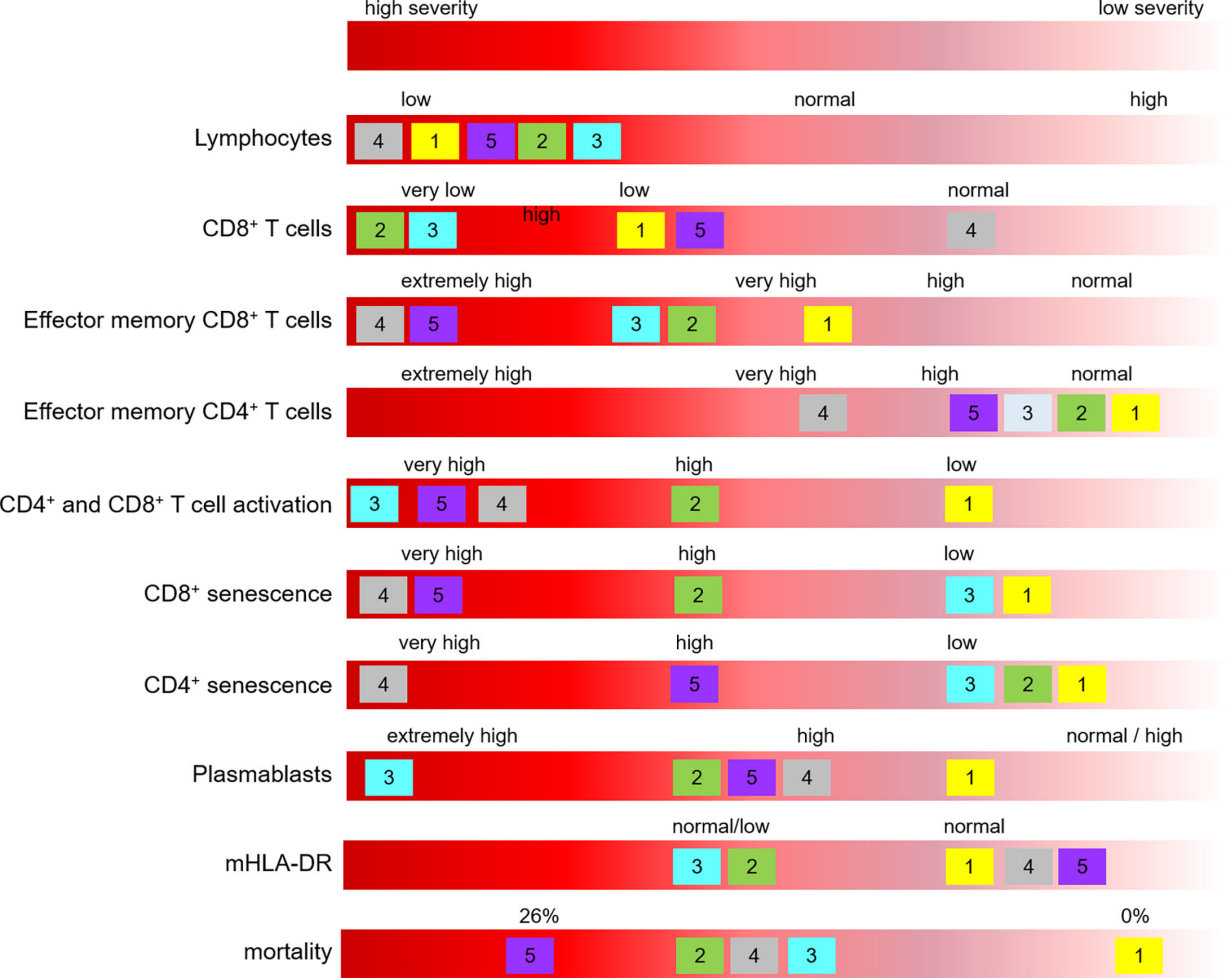
Main characteristics defining the five clusters based on immunophenotypic profile. Clusters are identified with their number, and positioned according to the mean value of the corresponding parameter.

Concerning patient characteristics, there was no statistically significant difference among the clusters for sex, BMI, C-Reactive Protein (CRP), classes of severity, O_2_ requirement and ICU admission. However, a significant difference was observed concerning mortality, with a higher death rate in clusters 2 and 5 compared with the other clusters (**Table 5**). The main characteristics defining the five clusters based on immunophenotypic profile are shown in **Table 6** and **Figure 3**.

**Table 5:** Overall comparison of clinical and biological characteristics between the clusters.

	Cluster 1	Cluster 2	Cluster 3	Cluster 4	Cluster 5	p-value
Men, n (%)	24 (58.5%)	13 (56.5%)	14 (66.7%)	12 (70.6%)	11 (47.8%)	0.630^1^
BMI, kg/m^2^, mean (sd) (n=94)	26.5 (6.3)	28.4 (4.7)	28.6 (5.8)	27.5 (7.2)	27.2 (6.2)	0.745^2^
CRP, mg/L, median [IQR] (n=99)	41 [18;100]	95 [31;155]	84 [18;131]	55.5 [19.5;121]	62.5 [48;151.5]	0.641^3^
Mortality, dead, n (%)	0 (0%)	4 (17.4%)	2 (9.5%)	2 (11.8%)	6 (26.1%)	0.005^1^ cluster 1 against others; 0.006^1^ clusters 2 and 5 against others
Severe Covid19^4^, n (%)	18 (43.9%)	14 (60.9%)	16 (76.2%)	9 (52.9%)	11 (47.8%)	0.154^1^
ICU admission, n (%)	11 (26.8%)	8 (34.8%)	12 (57.1%)	5 (29.4%)	5 (21.7%)	0.121^1^
Oxygen requirement, n (%)	27 (65.8%)	17 (73.9%)	16 (76.2%)	14 (82.3%)	13 (56.5%)	0.426^1^
Corticosteroid treatment, n (%)	12 (29.3%)	13 (56.2%)	13 (61.9%)	7 (41.2%)	6 (26.1%)	0.033^1^

^1^Fisher exact test.

^2^F-test Anova due to normality distribution.

^3^Kruskal Wallis test due to non-normality distribution.

^4^Severe Covid19 defined as: 0_2_>2L/min, ICU admission, LTE, decease.

**Table 6:** Major characteristics of the five clusters of patients.

	Cluster 1	Cluster 2	Cluster 3	Cluster 4	Cluster 5
**Lymphocytes G/L**	low	low	low	low	low
**CD8^+^ T cells**	low	very low	very low	normal	low
**Effector CD8^+^ T cells**	high	very high	very high	extremely high	extremely high
**Effector CD4^+^ T cells**	normal	normal	normal	high	normal
**CD4^+^ and CD8+ activation**	low activation	high	very high	very high	very high
**CD8^+^ senescence**	low senescence	high	low senescence	very high	very high
**CD4^+^ senescence**	low senescence	low senescence	low senescence	very high	high
**Plasmablasts**	normal/high	high	extremely high	high	high
**mHLA-DR**	normal	normal/low	normal/low	normal	normal
**Mortality^1^**	0	17.4	9.5	11.1	26.1

^1^p=0.005 cluster 1 against others; p= 0.006 clusters 2 and 5 against others.

## Discussion

This study had the objective of performing a fine analysis of lymphocyte subsets in SARS-CoV-2 infected patients hospitalized in Grenoble Alpes University Hospital between March and September 2020. For this exploratory study, an unsupervised statistical method, called hierarchical ascendant classification (HAC), was chosen. By using this method, we could classify the patients in different clusters on the basis of immunophenotypic data and age of patients, only. The HAC method establishes the ideal number of clusters; clinical data do not interfere upstream in the cluster definition. Thus, this method identified five different clusters of patients. Once the clusters were identified, we examined whether there was a biological and clinical difference between the clusters.

Our results strengthen previous studies showing heterogeneous profiles of SARS-CoV-2 infected patients obtained by unsupervised clustering and pointing out that disease severity may be associated with different profiles of immune response (8, 15–18). Our study focuses on lymphocyte subpopulations, in patients recruited the day of their admission to the hospital and not treated yet. Interestingly, phenotypic clusters of immune response did not exhibit statistically significant differences neither concerning sex, BMI and CRP (parameters that are usually associated with a more severe outcome), nor with high-flow oxygen requirement and ICU admission; however, different mortality outcome could be pointed out.

Even if some characteristics were similar among the different clusters, such as lymphopenia, and an elevated level of effector CD8^+^CCR7^-^ T cells, some other characteristics were very different: low lymphocyte activation and senescence in cluster 1, extremely elevated level of plasmablasts in cluster 3, high CD4^+^ and CD8^+^ T-cell activation in clusters 3, 4 and 5, and high CD8^+^ T-cell senescence in clusters 2, 4 and 5. The expansion of plasmablasts and plasma cells in some SARS-CoV-2 infected patients has been described (2, 9). Of note, the expression of HLA-DR molecules on circulating monocytes was in normal range in the study population, indicating that patients were mainly in a status of immunocompetence. Immunosuppression status which has been described in some studies (8) has been observed mainly in patients hospitalized in ICU.

Overall, exhibiting low levels of T-cell activation seems to be associated to a better disease outcome, as described in (9); on the other hand, exhibiting profound CD8^+^ T-cell lymphopenia, a high level of CD4^+^ and CD8^+^ T-cell activation and a high level of CD8^+^ T-cell senescence seems to be globally associated with a higher mortality outcome. The phenotype of exhausted T cells in SARS-CoV-2 infected patients has been described, with the expression of senescence and exhaustion markers such as CD57, PD-1 and CTLA-4 (17). In severe COVID-19 cases, the production of pro-inflammatory cytokines, including IL-1β, IL-6 and TNF-α, is increased leading to the generation of cytokine storm, inducing futher unfovarable outcome and may eventually lead to lymphopenia (19, 20).

In our study, we followed the classification of patients as severe if they required an oxygen therapy > 2 L/min; however many degrees of severity can exist in this group of patients. The lack of informations regarding oxygen therapy strategy, such as oxygen masks, CPAP or mechanical ventilation is a limitation of our study. Patients of our study were all hospitalized and recruited between March and September 2020. Therefore, we described here the immunophenotypic subset profiles of only severe cases from the first wave. It would be interesting to explore the profiles of not hospitalized patients with mild pathology, and those of patients infected with new circulating SARS-CoV-2 variants.

Our study suggests that some lymphocyte parameters might be useful to physicians to better characterize patients at hospital admission; in particular, identification of patients with potential mild (with low levels of T-cell activation) or very serious (with profound CD8^+^ T-cell lymphopenia, a high level of CD4^+^ and CD8^+^ T-cell activation and a high level of CD8^+^ T-cell senescence) evolution of the pathology could be helpful in order to treat them earlier and more appropriately. In this perspective, specific studies evaluating T-cell activation and senescence in a longitudinal patient follow-up are certainly needed.

## Data Availability Statement

The original contributions presented in the study are included in the article/**Supplementary Material**. Further inquiries can be directed to the corresponding author.

## Ethics Statement

The studies involving human participants were reviewed and approved by the Comité de Protection des Personnes Ile de France II. The patients provided written informed consent or non opposition, in accordance with the national legislation and the institutional requirements.

## Author Contributions

Funding acquisition: AG, OE, GC, PA. Immunological analysis: M-CJ, TR, MP. Methodology: MR. Statistical analysis: CL, AV, J-LB. Collection of patients’ samples and clinical information: OE, MM, AG. Writing original draft: GC, M-CJ. Review manuscript: M-CJ, J-YC, PP, CD-P, FD. All authors contributed to the article and approved the submitted version.

## Funding

This work was supported by funding from the Fondation Université Grenoble Alpes (project Biomarqueurs COVID-19).

## Conflict of Interest

The authors declare that the research was conducted in the absence of any commercial or financial relationships that could be construed as a potential conflict of interest.

## Publisher’s Note

All claims expressed in this article are solely those of the authors and do not necessarily represent those of their affiliated organizations, or those of the publisher, the editors and the reviewers. Any product that may be evaluated in this article, or claim that may be made by its manufacturer, is not guaranteed or endorsed by the publisher.
