# Supplementary material for: Fine Analysis of Lymphocyte Subpopulations in SARS-CoV-2 Infected Patients: Differential Profiling of Patients With Severe Outcome
Source: Front Immunol. 2022 Jul 15;13:889813. doi: 10.3389/fimmu.2022.889813 (PMC9335884; doi:10.3389/fimmu.2022.889813)
Supplement: Supplementary file 1 [file DataSheet_1.pdf]

Supplementary Material

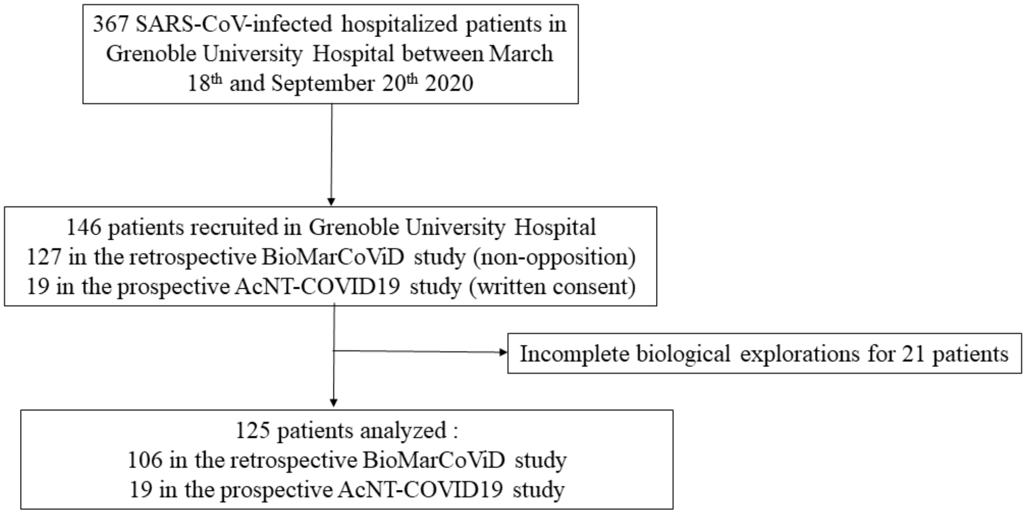

Supplementary Figure 1. Enrollment and inclusion of the patients.

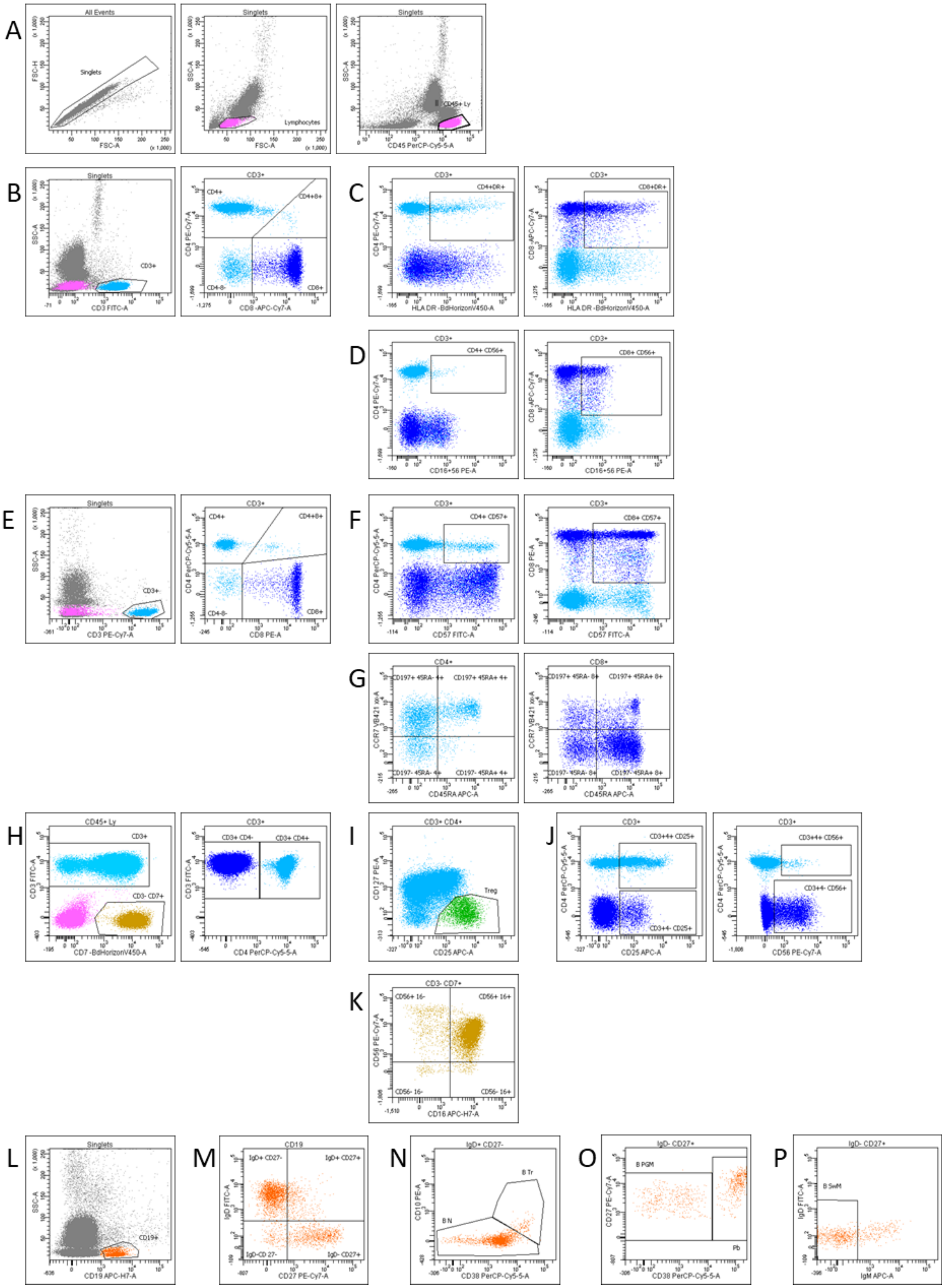
A
B
C
D
E
F
G
H
I
J
K
L
M
N
O
P

Supplementary Figure 2. Gating strategy for lymphocyte subset analysis.

Tube 1 (A to D). A. Selection of lymphocytes within singlets, FSC low SSC low and CD45 high cells. B. Selection of CD3+ T-cells, and CD4+ and CD8+ within CD3+ cells. C. Quantification of HLA-DR+ cells within CD4+ and CD8+ T-cells. D. Selection of CD56+ cells within CD4+ and CD8+ T-cells
Tube 2 (E to G). E. Selection of CD3+ T-cells within singlets, and CD4+ and CD8+ within CD3+ T-cells. F. Quantification of CD57+ cells within CD4+ and CD8+ T-cells. H. Quantification of naïve (CD45RA+ CD197+), central memory (CD45RA- CD197+), and effector memory (CD45RA+/- CD197-) CD4+ and CD8+ T-cells.
Tube 3 (H to K). H. Selection of CD3+ T-cells, CD7+ CD3- NK cells, CD3+ CD4+ T-cells and CD3+ CD4- (= CD8 T-cells) within CD3+ T-cells. I. Quantification of CD4+ $CD25_{high}$ $CD127_{low}$ T_{reg} within CD3+ T-cells. J. Quantification of CD25 and CD56 on CD4+ and CD4- T-cells. K. Quantification of CD56+ CD16+, CD56+ CD16-, CD56- CD16+ and CD56- CD16- within NK cells.
Tube 4 (L to P). L. Selection of CD19+ B-cells. M. Separation of 4 subsets according to IgD and CD27, among them IgD+ CD27+ natural memory B-cells. N. Within IgD+ CD27-, quantification of transitional CD38high CD10+ B-cells and CD38+/- CD10- naïve B-cells. O. Within IgD- CD27+, quantification of post-germinal memory CD38+/- B-cells and CD38 high plasmablasts. P. Within post-germinal memory B-cells, quantification of switch IgM negative B-cells.

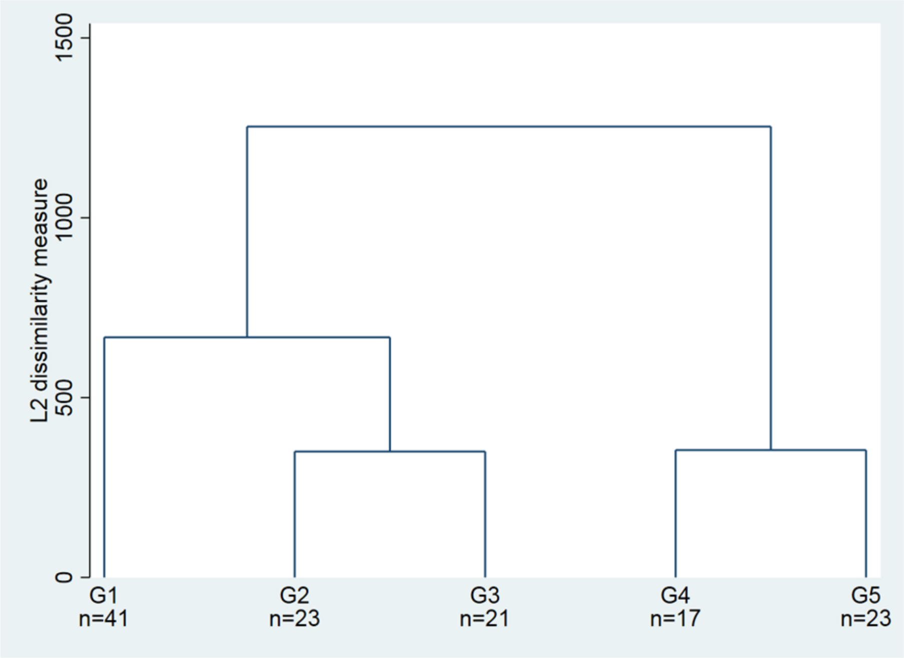

Supplementary Figure 3. Clustering of the patients.

Characteristics	Class of severity
no O_2 requirement	mild disease
$O_2 \leq 2L/min$	mild disease
$O_2 > 2L/min$	severe disease
ICU admission	severe disease
LTE	severe disease
decease	severe disease

LTE : Limitation of Therapeutic Effort

Supplementary Table 1. Classification of severity.

CD	Fluorochrome	Clone	Isotype	Supplier	Catalogue N°
CD3	FITC	SK7	Ms IgG1, κ	BD Biosciences	644611
CD4	PE-Cy7	SK3	Ms IgG1, κ	BD Biosciences	644611
CD8	APC-H7	SK1	Ms IgG1, κ	BD Biosciences	644611
CD19	APC	SJ25C1	Ms IgG1, κ	BD Biosciences	644611
CD16	PE	B73.1	Ms IgG1, κ	BD Biosciences	644611
CD56	PE	NCAM16.2	Ms IgG2b, κ	BD Biosciences	644611
CD45	PerCP	2D1	Ms IgG1, κ	BD Biosciences	644611
HLA-DR	V450	L243	Ms IgG2a, κ	BD Biosciences	655874
CD8	PE	B9.11	Ms IgG1	Beckman Coulter	A07757
CD4	PerCP-Cy5.5	SK3	Ms IgG1, κ	BD Biosciences	332772
CD3	PE-Cy7	UCHT1	Ms IgG1	Beckman Coulter	737657
CD45RA	APC	HI100	Ms IgG2b, κ	BD Biosciences	550855
CD197	VB421	150503	Ms IgG2a	BD Biosciences	562555
IgD	FITC	polyclonal	rabit anti-human	Agilent technologies	F018901
CD10	PE	HI10a	Ms IgG1, κ	BD Biosciences	332776
CD38	PerCP-Cy5.5	HIT2	Ms IgG1, κ	BD Biosciences	551400
CD27	PE-Cy7	1A4CD27	Ms IgG1	Beckman Coulter	B49205
IgM	APC	G20-127	Ms IgG1, κ	BD Biosciences	551062
CD19	APC-H7	SJ25C1	Ms IgG1, κ	BD Biosciences	641395
CD127	PE	HIL-7R-M21	Ms IgG1, κ	BD Biosciences	557938
CD56	PE-Cy7	N901 (NKH-1)	Ms IgG1	BD Biosciences	A21692
CD25	APC	2A3	Ms IgG1, κ	BD Biosciences	340907
CD16	APC-H7	3G8	Ms IgG1, κ	BD Biosciences	560195
CD7	V450	T701	Ms IgG1, κ	BD Biosciences	642916
CD45	V500	HI30	Ms IgG1, κ	BD Biosciences	560777

Supplementary Table 2. Characteristics of antibodies.

	Mean	sd	Min.	lower quart.	Median	upper quart.	Max.
Leucocytes, G/L	6.9	3.5	2	4.3	6.2	8.6	20.2
Lymphocytes, G/L	1.0	0.5	0.2	0.6	0.9	1.3	2.8
Lymphocytes, %	18	12	2	9	15	23	61
**T-cell subsets**							
Total $CD3^+$ T cells, G/L	0.7	0.4	0.1	0.4	0.6	0.9	2.3
Total $CD4^+$ T cells , G/L	0.5	0.3	0.1	0.2	0.4	0.6	1.5
Total $CD8^+$ T cells, G/L	0.2	0.2	0.0	0.1	0.2	0.3	1.2
Total $CD3^+$ T cells, %	70	11	43	62	72	79	90
Total $CD4^+$ T cells, %	45	12	18	36	46	54	70
Total $CD8^+$ T cells, %	21	11	3	13	20	26	69
$CD4^+/CD8^+$	3.0	2.6	0.3	1.6	2.3	3.6	21
Naive $CD4^+$ T cells, %	45	17	3	33	46	56	83
Central memory $CD4^+$ T cells, %	36	13	1	26	36	44	69
Effector $CD4^+$ T cells, %	19	13	3	12	16	24	71
Naive $CD8^+$ T cells, %	25	18	2	10	22	36	70
Central memory $CD8^+$ T cells, %	11	9	0	5	8	15	62
Effector $CD8^+$ T cells, %	64	20	20	49	64	81	98
Regulatory T cells, %	8	3	2	5	7	9	20
$CD4^-CD8^-/CD3^+$, %	4	3	0	2	3	4	13
**T-cell activation markers**							
$HLA\text{-}DR^+/CD4^+$, %	12	8	3	6	9	15	53
$HLA\text{-}DR^+/CD8^+$, %	35	18	7	22	31	46	79
**T-cell senescence markers**							
$CD57^+/CD4^+$, %	5	9	0	1	2	6	63
$CD57^+/CD8^+$,%	29	17	0	14	25	44	78
**B-cell subsets**							
Total B cells, G/L	0.1	0.1	0.0	0.1	0.1	0.2	0.6
Total B cells, %	14	8	2	8	12	18	49
Transitional B cells, %	4	3	0	1	3	6	19
Naive B cells, %	49	19	1	39	50	63	84
Natural memory B cells, %	10	9	0	4	7	11	55
Post germinal memory B cells, %	16	13	2	9	13	19	88
Plasmablasts, %	13	15	0	3	7	18	86
**NK-cell subsets**							
Total NK cells, G/L	0.1	0.1	0.0	0.1	0.1	0.2	0.6
Total NK cells, %	15	8	2	8	14	19	40
Cytotoxic NK cells, %	88	11	2	85	91	94	97
Immunomodulatory NK cells, %	4	6	0	1	2	4	47
Inflammatory NK cells, %	7	9	0	3	4	8	92
**Monocytes**							
Total monocytes, %	7	3	1	5	7	10	16
Non-conventional monocytes, %	13	9	1	7	11	18	45
mHLA-DR, AB/C[1]	36847	19587	7765	19850	35952	50973	88031

[1]number of antibodies fixed per cell

Supplementary Table 3. Quantification of cellular subsets.

	Cluster 1	Cluster 2	Cluster 3	Cluster 4	Cluster 5	p-value
Leucocytes, G/L	5.7 [3.7;8.2]	6.3 [4.6;10.3]	8.5 [5.9;10.8]	5.4 [4.1;7.8]	6.1 [3.9;7.1]	0.089[1]
Lymphocytes, G/L	0.9 [0.7;1.5]	0.9 [0.6;1.4]	0.6 [0.4;1.2]	1.2 [0.7;1.5]	0.8 [0.6;1.3]	0.27[1]
Lymphocytes, %	18 [12;28]	1 4 [7;23]	7 [6;15]	19 [11;24]	17 [10;23]	0.018[1]
T-cell subsets						
Total $CD3^+$ T cells, G/L	0.7 [0.5;1.1]	0.6 [0.4;0.9]	0.4 [0.3;0.7]	0.8 [0.6;1]	0.6 [0.4;0.9]	0.11[1]
Total $CD4^+$ T cells , G/L	0.5 [0.3;0.8]	0.4 [0.2;0.6]	0.3 [0.2;0.5]	0.4 [0.2;0.5]	0.4 [0.3;0.6]	0.049[1]
Total $CD8^+$ T cells, G/L	0.2 [0.1;0.3]	0.1 [0.1;0.2]	0.1 [0.1;0.2]	0.4 [0.2;0.5]	0.2 [0.1;0.2]	0.001[1]
Total $CD3^+$ T cells, %	72 [67;81]	63 [55;71]	67 [59;74]	77 [74;82]	74 [66;83]	p<0.001[1]
Total $CD4^+$ T cells, %	52 (10)	43 (10)	43 (12)	32 (9)	48 (10)	p<0.001[2]
Total $CD8^+$ T cells, %	17 (8)	17 (7)	19 (7)	38 (14)	22 (8)	p<0.001[2]
$CD4^+/CD8^+$	3.1[2;4.9]	2.8 [1.7;3.5]	2 [1.5;3.4]	0.9 [0.8;1.5]	1.9 [1.6;3.6]	p<0.001[1]
Naive $CD4^+$ T cells, %	53 (16)	36 (12)	47 (18)	25 (10)	51 (13)	p<0.001[2]
Central memory $CD4^+$ T cells, %	34 (12)	49 (11)	32 (8)	36 (11)	28 (11)	p<0.001[2]
Effector $CD4^+$ T cells, %	12 [8;16]	14 [11;18]	15 [12;18]	35 [32;42]	19 [12;26]	p<0.001[1]
Naive $CD8^+$ T cells, %	46 [32;55]	21 [15;27]	22 [15;33]	7 [5;11]	8 [6;14]	p<0.001[1]
Central memory $CD8^+$ T cells, %	11 [7;19]	15 [10;18]	6 [5;12]	5 [3;8]	4 [3;7]	p<0.001[1]
Effector $CD8^+$ T cells, %	44 [36;50]	64 [57;70]	71 [58;75]	87 [81;91]	85 [82;90]	p<0.001[1]
Regulatory T cells, %	7 [5;8]	9 [7;10]	8 [6;10]	7 [5;9]	7 [6;8]	0.049[1]
$CD4^-CD8^-/CD3^+$, %	2 [2;4]	3 [2;5]	2 [2;3]	4 [2;4]	2 [1;6]	0.463[1]
T-cell activation markers						
$HLA\text{-}DR^+/CD4^+$, %	6 [5;8]	9 [7;11]	14 [9;19]	14 [12;18]	13 [8;18]	p<0.001[1]
$HLA\text{-}DR^+/CD8^+$, %	18 [14;27]	31 [26;40]	55 [33;62]	37 [24;50]	53 [31;63]	p<0.001[1]
T-cell senescence markers						
$CD57^+/CD4^+$, %	1 [1;2]	1 [1;3]	2 [1;4]	16 [11;23]	6 [1;9]	p<0.001[1]
$CD57^+/CD8^+$, %	14 [10;19]	32 [26;44]	18 [13;23]	50 [47;54]	44 [31;54]	p<0.001[1]
B-cell subsets						
Total B cells, G/L	0.1 [0.1;0.2]	0.1 [0.1;0.2]	0.1 [0.1;0.2]	0.1 [0.0;0.1]	0.1 [0.0;0.1]	0.049[1]
Total B cells, %	12 [8;17]	17 [12;23]	17 [10;24]	10 [7;11]	9 [6;12]	0.001[1]
Transitional B cells, %	4 [2;6]	4 [2;6]	1 [1;4]	5 [2;6]	1 [1;3]	p<0.001[1]
Naive B cells, %	58 (16)	55 (14)	31 (17)	53 (12)	43 (22)	p<0.001[2]
Natural memory B cells, %	8 [4;12]	8 [4;10]	6 [3;21]	9 [5;14]	6 [4;9]	0.794[1]
Post germinal memory B cells, %	12 [8;18]	14 [9;18]	9 [7;16]	13 [9;19]	15 [11;35]	0.056[1]
Plasmablasts, %	4 [2;11]	7 [3;12]	29 [23;42]	5 [3;10]	6 [3;14]	p<0.001[1]
NK-cell subsets						
Total NK cells, G/L	0.1 [0.1;0.2]	0.1 [0.1;0.2]	0.1 [0.0;0.1]	0.1 [0.1;0.2]	0.1 [0.1;0.2]	0.57[1]
Total NK cells, %	12 [7;18]	15 [9;25]	14 [8;21]	12 [10;18]	14 [6;21]	0.595[1]
Cytotoxic NK cells, %	89 [85;94]	89 [79;92]	90 [88;94]	92 [85;96]	92 [90;94]	0.105[1]
Inflammatory NK cells, %	2 [1;4]	3 [1;7]	2 [1;4]	2 [1;3]	2 [2;5]	0.661[1]
Immunomodulatory NK cells, %	5 [4;9]	6 [4;13]	4 [3;8]	4 [3;7]	4 [2;6]	0.206[1]
Monocytes						
Total monocytes, %	8.0 (3.6)	7.5 (3.3)	7.1 (2.7)	7.0 (3.0)	7.2 (3.3)	0.779[2]
Non-conventional monocytes, %	12 [7;19]	11 [7;14]	5 [3;12]	13 [10;17]	14 [10;26]	0.003[1]
mHLA-DR, AB/C[3]	42044 (17038)	25218 (17485)	21009 (9786)	44608 (20302)	47150 (19101)	0.004[2]
Other						
Age	56 [44.6;72.1]	70 [56.6;75.6]	71 [62.3;80.6]	72 [64.1;82.5]	79 [70.8;89.3]	p<0.001[1]

[1] p-value from Kruskal Wallis test due to non-normality distribution; median [IQR]
[2] p-value from F-test Anova due to normality distribution; mean (SD)
[3] number of antibodies fixed per cell

Supplementary Table 4. Overall comparison of cellular subpopulations between the clusters.
